# Monthly Incidence Rates of Abusive Encounters for Canadian Family Physicians by Patients and Their Families

**DOI:** 10.1155/2010/387202

**Published:** 2010-11-11

**Authors:** Baukje (Bo) Miedema, Ryan Hamilton, Sue Tatemichi, Anita Lambert-Lanning, Francine Lemire, Donna Manca, Vivian R. Ramsden

**Affiliations:** ^1^Family Medicine Teaching Unit, Dr. Everett Chalmers Regional Hospital, Dalhousie University, 700 Priestman Street, P.O. Box 9000, Fredericton, NB, Canada E3B 5N5; ^2^National Research System (NaReS), College of Family Physicians of Canada, 2630 Skymark Avenue, Mississauga, ON, Canada L4W 5A4; ^3^Membership Department, College of Family Physicians of Canada, 2630 Skymark Avenue, Mississauga, ON, Canada L4W 5A4; ^4^Department of Family Medicine, University of Alberta, 901 College Plaza, Edmonton, AB, Canada T6G 2C8; ^5^West Winds Primary Health Centre, Department of Academic Family Medicine, College of Medicine, University of Saskatchewan, 3311 Fairlight Drive, Saskatoon, SK, Canada S7M 3Y5

## Abstract

*Objective*. The goal of this study was to examine the monthly incidence rates of abusive encounters for family physicians in Canada. *Methods*. A 7-page cross-sectional survey. *Results*. Of the entire study sample (*N* = 720), 29% of the physicians reported having experienced an abusive event in the last month by a patient or patient family member. Abusive incidents were classified as minor, major, or severe. Of the physician participants who reported having been abused, *all* reported having experienced a minor event, 26% a major, and 8% a severe event. Of the physicians who experienced an abusive event, 55% were not aware of any policies to protect them, 76% did not seek help, and 64% did not report the abusive event. *Conclusion*. Family physicians are subjected to significant amounts of abuse in their day-to-day practices. Few physicians are aware of workplace policies that could protect them, and fewer report abusive encounters. Physicians would benefit from increased awareness of institutional policies that can protect them against abusive patients and their families and from the development of a national policy.

## 1. Introduction

Although data from the 2007 National Physicians' Survey (NPS) indicates that most family physicians are satisfied with their clinical practices, studies in several countries have reported that violence in family practice towards physicians is a serious problem [1–11]. In New Zealand, it was reported that on an annual basis, 15% of general practioners were verbally abused, 4% were assaulted, and 2% were stalked [8]. In an Australian study, it was reported that, in the previous year, 64% of general practitioners experienced violence in their practice. The majority of physicians reported having experienced “low levels” of violence; however 9% reported having been sexually harassed, and 3% reported being physically harmed. Australian research indicates that younger physicians and those working in “after hours” settings experience the highest levels of workplace abuse [7]. Verbal abuse or disrespectful behavior is the most common type of abuse; in Japan, 31.8% of physicians reported that they had been verbally abused in the last 6 months [12, 13]. Verbal threats are a more serious form of verbal abuse. A study in the USA reported that in any given year, 75% of all US emergency department (ED) physicians experienced at least one verbal threat [14]. 

Although violent abusive encounters are rare, minor abusive encounters also may have a negative impact on victims [9]. For example, of the ED workers who had experienced violence at work, almost half reported that their job performance was impaired for a short time whereas others reported being affected for up to a week after the abusive experience [15]. Long wait times for appointments, physician shortages, limited resources, and general stress can all lead to patient frustration and subsequent violence [16]. 

Researchers in Australia and the UK have recently examined violence against physicians however few have examined the issue in Canada [1, 17]. Canadian studies that have been undertaken are at least a decade old and are limited in scope. A 1993 study examining sexual harassment among female physicians in Ontario revealed that 77% of the respondents indicated that they had been sexually harassed at least once in their careers [18, 19]. There are reports of physical abuse toward physicians in training [20], and a family physician from Alberta went public with her experience of being stalked by a patient [21]. 

Many countries are developing policies to protect physicians from violent patients [22–25], but Canada does not have a national policy. The current study is the first of its kind in Canada to examine violence in the workplace of family physicians. We have recently published a paper on the career prevalence of workplace violence among Canadian family physicians [26]. This paper will focus on the *monthly incidence* rates of violence against family physicians and the characteristics of the most common perpetrators of the violence.

## 2. Methods

The study employed a mixed-methods approach, using a cross-sectional survey to collect quantitative data of a sample of 3802 members of the College of Family Physicians of Canada. Physicians who had experienced abuse in the previous year were invited to participate in a qualitative telephone interview. The survey was a modified survey developed by a research team from New Zealand [8]. Part I of the survey included demographic questions pertaining to gender, practice location, and type of practice. Part II included questions about the career prevalence and frequency of 14 different types of abusive encounters ranging from minor to severe. A 5-point Likert scale ranging from “never” to “very often” regarding the frequency of the abuse was utilized. Part III asked about the monthly incidence of abusive encounters by perpetrators ranging from minor to severe. Part IV asked questions regarding policy and actions. The survey was seven pages in length, and face validity was obtained through expert reviews by several family physicians in Fredericton, New Brunswick. 

The College of Family Physicians of Canada's (CFPC's) National Research System (NaReS) operationalized the survey for this study using a modified Dillman approach. This survey was pilot tested for NaReS by four English speaking and four French speaking family physicians across the country. Some minor changes were made to the English survey while for the French survey several terms were altered to mirror the language used in the 2007 National Physician Survey (NPS) [11]. The pilot survey data was not included in the final sample. 

The survey was mailed to a random sample of family physicians who are members of the CFPC and self-identified as “active/practicing” family physicians. Members of the Family Physicians' Workplace Abusive and Violent Encounters (FPWAVEs) research team, retired, sustaining, specialist, and resident members were excluded. Research Ethics Board approval for the study was granted by Dalhousie University, the University of Alberta, and the University of Saskatchewan.

Most of the survey questions were nominal (demographic information or dichotomous (yes/no) in nature). The monthly incidence rate of abuse, the type of abuse, the characteristics of the abuser, and the characteristics of the victim were calculated. Summary statistics such as measures of central tendency and dispersion (Chi-squares) were calculated to describe the results.

## 3. Results

Of all the mailed surveys, (*N* = 3802) 20.3% were returned (*N* = 774). Four were returned blank. For the analysis of this paper, we removed 35 surveys because the physicians had indicated that the previous month had not been a regular work month. They had spent less time (<2 weeks) in the practice than usual. We also removed cases that had missing data on the monthly incident rate questions. The total number of surveys used for the analysis of this paper, the monthly incidence rates of abusive events in family practice, was 720.

### 3.1. Demographic Profile of All Participants

More female than male physicians participated in the study. One-fifth of the participants identified themselves as belonging to a visible minority, and 3% identified themselves as being homosexual or bisexual. The private office was the main practice setting in urban/suburban settings. The average number of years in practice was 14.6 (SD 10.19) the average number of hours worked per week was 46.5 (SD 15.64), and the average of weekly clinical hours worked was 36.7 (SD 12.94) (Table 1).

### 3.2. Monthly Incidence Rate of Abusive Events

Twenty-nine percent (*N* = 209) of the physicians reported that they had experienced abuse by either a patient or a family member of a patient in the last month. Many physicians reported having experienced multiple abusive encounters in the last month with an average of 2.47 (SD 2.37) incidents for a total number of 540 incidents. Of these abusive incidents, 420 (78%) were committed by patients and 120 (22%) by family members of patients. 

In the survey, the abusive events were categorized into 13 descriptive headings, ranging in severity from minor, such as “disrespectful behavior,” to severe, such as “stalking.” We have collapsed the 13 abusive headings into three major categories: minor, major, and severe incidents (Table 2).

All physicians who reported abusive incidents noted that the majority of the events were minor; however one quarter of the physicians reported their abusive encounters as major, and one in twelve reported that the abuse was severe (Table 3).

### 3.3. Characteristics of Abused Physicians

For a profile of physicians who encountered abusive patients and/or family members of patients, we used Chi-square calculations to examine statistically significant relationships between the dependent and independent variables and between physicians who reported abuse and physicians who did not. Slightly more female physicians reported being abused than males, 30% versus 28% (*P*  value = NS). There were also no statistically significant differences between Heritage Identification variables. Practice location was a significant factor in the incidence of abuse, with 59% of physicians working in the ED reporting being involved in an abusive incident in the last month (*P*  value ≤ .001). This was the highest incidence rate reported in the study. The next highest practice setting for abusive experiences was that of private practice (28%). Physicians in small towns experienced the highest levels of abuse at 34% although it was not statistically significant.

### 3.4. Profile of an Abuser

More male than female patients are abusive; however more female family members of patients are abusive than male family members of patients. Almost one-fifth of the abusive patients were intoxicated (19%), but only 5% of abusive family members of patients were considered intoxicated. Physicians reported that one-third of the abusive patients and 14% of the abusive family members were repeat abusers. Abused physicians reported that one-third of abusive patients had mental health issues, but the majority did* not*. Abusive events occurred most commonly in the afternoon (42%), and most took place during a face-to-face encounter (Table 4).

Of the 209 family physicians who reported having been abused in the last month, 17 (8%) reported having encountered severe abuse representing 27 incidents, 14 (52%) incidents were attempted assault, 6 (22%) assaults, 2 (7%) assaults resulting in injury, 3 (11%) sexual assaults, and 2 (7%) stalking incidents. Patients were responsible for all of the severe abusive incidents.

### 3.5. Action by the Abused Physicians

Of the physicians who experienced an abusive incident in the last month, 55% were not aware of any policies to “protect you from abuse/violence in the workplace,” 76% neither sought help nor “accessed any resources regarding workplace abuse/violence,” and 64% have never reported an abusive incident. The majority of physicians were not aware of institutional policies they could draw upon to aid them.

## 4. Discussion

Canadian family physicians in active practice are subjected to regular abuse from their patients or family members of their patients. Although the majority of abusive encounters can be characterized as minor, one-third of physicians reported having been subjected to major or severe abusive events. Some of the study participants reported that the abusive persons were intoxicated or had mental health problems, and therefore it could be argued that these abusers could not be held responsible for their actions. However, the *majority* of abusers were not so affected. 

Our study confirms prior research which has found that certain work settings are associated with higher rates of abuse than others. Working in the ED significantly increased the risk of being abused for practitioners in our study. However, other practice settings are not immune from abusive incidents perpetrated by patients or their family members. The majority of abusive incidents are verbal abuse; nevertheless, an alarming number of family physicians experience severe abuse that can lead to injury or involve the physicians' families (stalking). 

Our data is similar to data from other countries. For example, studies in Australia, New Zealand, the United Kingdom, Japan, and the USA also indicate that verbal abuse is the most prevalent [7, 12, 27]. In Japan, over 31.8% of physicians had experienced verbal abuse in the last six months, while we report that just under 30% experienced this in the last month [12]. In New Zealand, 4% of general practitioners were assaulted, and 2% were stalked in the previous year. Our numbers are similar to their study but cannot really be compared due to the different study parameters and time frames examined [8]. What is unique about our study is that it is national in scope and also examines perpetrator and victim characteristics from the perspective of the affected physicians. To our knowledge, no other studies have examined the characteristics of patients and family of patients who are abusive toward their family physicians. Except for some family physicians who practice in EDs, no particular characteristic made physicians more or less vulnerable to abuse. For patients and family members of patients who are abusive, we now know that the majority do not have mental health issues nor are intoxicated. 

According to the Canadian Medical Association 2009 Report [28], Canada has 34,403 family physicians. If we extrapolate our data to all family physicians in Canada, almost 10,000 family physicians are abused every month by a patient or a family member of a patient and 700 experience severe abuse that may include physical injury and stalking. Although physician abuse is a serious issue, the 2007 NPS data indicated that 85.3% of family physicians, and general practitioners are very or somewhat satisfied with the relationships with their patients [11]. Thus it seems that negative experiences with abusive patients or family of patients do not translate into negative feelings towards all patients. 

According to our study, more than half of the family physicians in Canada who had experienced abuse were not aware of any policies that could protect them from abuse in the workplace. Perhaps as a result of the lack of knowledge and the lack of a national policy, few sought help. Is it possible that the lack of knowledge regarding regional or national policies about workplace abuse encourages an attitude among family physicians that abusive encounters are the norm? Other countries such as Australia and the United Kingdom have national policies that assist physicians to protect themselves against violent patients [24, 25].

One of the potentially significant issues of this violence in the workplace of family physicians is that it may influence family physicians to provide services in certain practice settings, to the exclusion of environments that are associated with more abusive encounters with patients. In an Australian study regarding violence and “after hours” care setting, Magin et al. [7] concluded that when physicians feel under threat, they will withdraw their services. Fernandes and colleagues [15] also reported that ED physicians demonstrate a significant reduction in job satisfaction and an increased amount of stress due to violent patient behavior toward physicians. Ignoring violence in the workplace of family physicians in Canada can have long-term negative consequences for the individual physician and for the health care system as a whole.

## 5. Limitations

We acknowledge that the response rate for this survey was low; however, the monthly incidence rate (last month) reduced the recall bias present in studies that use different time frames. As with all survey studies, this study was based on self-report and the abusive encounters were not corroborated with administrative data. We have no reason to doubt the responses of family physicians who took the time to complete this lengthy survey and who provided additional responses to some of the questions. Another potential limitation involves the classification of abusive behaviours into “minor”, “major”, and “severe” categories. We acknowledge that these labels appear to be reflective of the impact of abuse; however, we did not request from the participants to label the nature of the abusive incidents. Thus, our classification reflects researcher defined criteria.

## 6. Conclusion

Canadian family physicians are generally pleased with their interactions with patients and their families; however these interactions are not always pleasant or safe for family physicians. Unfortunately, family physicians are often victims of abuse—verbal, physical, sexual, and psychological—from patients or the family of patients. In this comprehensive pan-Canadian study, we have demonstrated that all family physicians, regardless of their practice location, are at risk of abuse, and neither mental illness nor inebriation is the main factor in those abusive encounters. Minor abusive encounters are very common, and of significant concern is the number of major and severe abusive encounters that take place in the primary care setting. Fortunately, the negative experiences with abusive patients and/or their family members do not appear to reduce patient relationship satisfaction. Finally, Canadian medical associations need to develop national policies, just as in Australia and the United Kingdom, that assist family physicians in protecting them from abusive patients and their family members. Physicians, just like other frontline healthcare workers, are entitled to a safe workplace without violence.

## Figures and Tables

**Table 1:** Demographic information of 720 participants.

Variable	Number	(%)
Gender		
Male	320	(44.4%)
Female	400	(55.6%)
Heritage identification		
Caucasian	564*	(79.5%)
Minority	145	(20.5%)
Sexual orientation		
Heterosexual	685*	(97.0%)
Homosexual	17	(2.4%)
Bisexual	4	(0.6%)
Main patient care setting		
Private office	452*	(63.5%)
Emergency department	81	(11.4%)
Community clinic	63	(8.8%)
Academic	43	(6.0%)
Other	73	(10.2%)
Location of main practice setting		
Inner city	84*	(11.8%)
Urban/Suburban	415	(58.1%)
Small town	119	(16.7%)
Rural/Remote	94	(13.1%)

*Some categories have missing data.

**Table 2:** Categories of seriousness of levels of abuse.

*Disrespectful behavior:*	*Minor* Incidents
Abuser was rude and/or disrespectful.	
*Bullying: *	
Abuser was belittling or professionally humiliating.	
*Verbal anger:*	
Abuser was loud, angry, or insulting but NOT threatening.	
*Verbal threat:*	
Abuser was loud, angry, or insulting and threatening.	
*Humiliation:*	
Abuser insulted you, called you names, or gestured to you in a manner as to decrease your self-esteem or humiliate you.	

*Physical aggression:*	*Major* incidents
Abuser threw objects, slammed doors, kicked, or gestured but did NOT damage persons or property.	
*Destructive behavior:*	
Abuser broke or smashed objects and kicked or struck out and caused damage to possessions and property but NOT to any persons.	
*Sexual harassment:*	
Abuser spoke, looked, or gestured in a manner that you perceived as an unwanted sexual advance.	

*Assault:*	*Severe* incidents
Abuser hit, punched, kicked, pulled, or pinched you WITHOUT injury.	
*Assault Causing Injury:*	
Abuser hit, punched, kicked, pulled, or pinched you causing injury.	
*Attempted Assault:*	
Abuser broke, smashed, kicked, or struck out towards you but did NOT hit or harm you.	
*Sexual assault:*	
Abuser physically touched or assaulted you in a manner you perceived as unwanted and of a sexual nature.	
*Stalking:*	
Abuser monitored, followed, or stalked you.	

**Table 3:** Type of abusive incidents.

Type of abuse	Abusive incidents (*n*=209)*	
	*N*	%
Minor incidents	208	99.9%
Major incidents	55	26.0%
Severe incidents	17	8.0%

*Not mutually exclusive. A physician may have experienced multiple types of abuse in the last month.

**Table 4:** Characteristic of the abuser and the abusive event.

Characteristics of abuser/abuse	Patient perpetrator %	Family member perpetrator %
Gender		
Male	56.0%	33.7%
Female	44.0%	66.3%
Abuser was intoxicated		
Yes	18.8%	4.8%
No	81.2%	95.2%
Abuser victimized before		
Yes	35.6%	14.5%
No	64.4%	85.5%
Abuser mentally ill		
Yes	35.6%	NA
No	64.4%	NA
Abuse occurred in the		
Morning	26.2%	22.2%
Afternoon	42.1%	53.1%
Evening	22.4%	24.7%
Night	9.3%	0.0%
Abuse was conducted		
Face to face	90.8%	77.1%
By telephone	7.0%	20.5%
Through email	1.1%	2.4%
Through mail	1.1%	0.0%
